# Choice-Related Activity during Visual Slant Discrimination in Macaque CIP But Not V3A

**DOI:** 10.1523/ENEURO.0248-18.2019

**Published:** 2019-03-26

**Authors:** L. Caitlin Elmore, Ari Rosenberg, Gregory C. DeAngelis, Dora E. Angelaki

**Affiliations:** 1 Department of Neuroscience, Baylor College of Medicine, Houston, Texas 77030; 2 Department of Neuroscience, School of Medicine and Public Health, University of Wisconsin-Madison, Madison, Wisconsin 53705; 3 Department of Brain and Cognitive Sciences, Center for Visual Science, University of Rochester, Rochester, New York 14627

**Keywords:** 3D orientation, CIP, macaque, perception, slant, V3A

## Abstract

Creating three-dimensional (3D) representations of the world from two-dimensional retinal images is fundamental to visually guided behaviors including reaching and grasping. A critical component of this process is determining the 3D orientation of objects. Previous studies have shown that neurons in the caudal intraparietal area (CIP) of the macaque monkey represent 3D planar surface orientation (i.e., slant and tilt). Here we compare the responses of neurons in areas V3A (which is implicated in 3D visual processing and precedes CIP in the visual hierarchy) and CIP to 3D-oriented planar surfaces. We then examine whether activity in these areas correlates with perception during a fine slant discrimination task in which the monkeys report if the top of a surface is slanted toward or away from them. Although we find that V3A and CIP neurons show similar sensitivity to planar surface orientation, significant choice-related activity during the slant discrimination task is rare in V3A but prominent in CIP. These results implicate both V3A and CIP in the representation of 3D surface orientation, and suggest a functional dissociation between the areas based on slant-related choice signals.

## Significance Statement

Surface orientation perception is fundamental to visually guided behaviors such as reaching, grasping, and navigation. Previous studies implicate the caudal intraparietal area (CIP) in the representation of 3D surface orientation. Here we show that responses to 3D-oriented planar surfaces are similar in CIP and V3A, which precedes CIP in the cortical hierarchy. However, we also find a qualitative distinction between the two areas: only CIP neurons show robust choice-related activity during a fine visual orientation discrimination task.

## Introduction

Perception of three-dimensional (3D) surface orientation is essential for many visually guided behaviors. Electrophysiological studies have identified 3D orientation-selective neurons in multiple brain regions of nonhuman primates (Murata et al., 2000; Taira et al., 2000; Hinkle and Connor, 2002; Sugihara et al., 2002; Nguyenkim and DeAngelis, 2003; Liu et al., 2004; Durand et al., 2007; Sanada et al., 2012; Alizadeh et al., 2018). In particular, the caudal intraparietal area (CIP) represents all combinations of slant and tilt, two angular variables that specify the 3D orientation of a planar surface (Rosenberg et al., 2013). Anatomic as well as functional magnetic resonance imaging (MRI) data suggest that V3A, which precedes CIP in the visual hierarchy, may also contribute to 3D visual processing (Nakamura et al., 2001; Tsao et al., 2003). V3A neurons have two-dimensional orientation (Zeki, 1978a,b,c) and binocular disparity (Anzai et al., 2011) tuning, but their responses to 3D surface orientation have not been examined. Moreover, few studies have tested for functional correlations between neuronal activity and 3D orientation perception. Previous work indicates that reversible inactivation of CIP results in small but consistent deficits in a 3D curvature discrimination task (Van Dromme et al., 2016), and may produce a deficit in the ability to perform a delayed match-to-sample task in which planar tilt is coarsely manipulated (Tsutsui et al., 2001).

Here we measured the responses of V3A and CIP neurons to 3D surface orientation, as well as their functional correlations with behavior during a fine slant discrimination task. First, 3D surface orientation tuning was measured during a fixation task. The two areas were found to contain similar proportions of selective neurons, as well as similar degrees of selectivity. Second, neuronal activity was recorded while the monkeys viewed planar surfaces at different slants and reported the slant direction in a two-alternative forced-choice task. Receiver operating characteristic (ROC) analysis was used to quantify neuronal sensitivity and to assess choice-related activity (Celebrini and Newsome, 1994; Britten et al., 1996; Dodd et al., 2001; Nienborg and Cumming, 2006; Gu et al., 2007). In contrast to the similarity of stimulus selectivity in the two areas, significant choice-related activity was rare in V3A but prominent in CIP. To further dissociate the contributions of stimulus and choice to neuronal activity, we performed a partial correlation analysis to assess how much variance in the neuronal activity could be attributed to the stimulus and the choice (Zaidel et al., 2017). This analysis confirmed a similar degree of stimulus-related activity in the two areas, and much stronger choice-related activity in CIP than V3A. These results implicate both V3A and CIP in visual surface orientation processing, and demonstrate that binary decision signals during slant discrimination are carried by the most sensitive CIP (but not V3A) neurons.

## Materials and Methods

### Subjects and surgery

All surgeries and experimental procedures were approved by the Institutional Animal Care and Use Committees at Washington University in St. Louis and Baylor College of Medicine, and were performed in accordance with National Institutes of Health guidelines. Neuronal recordings were obtained from five hemispheres in three male rhesus monkeys (*Macaca mulatta*), denoted as monkeys N, P, and Z, weighing 4-5 kg at the start of the study. As previously described, the monkeys were chronically implanted with a lightweight plastic ring for head restraint, a recording grid, and scleral eye coils for monitoring binocular eye movements (CNC Engineering; Rosenberg et al., 2013). After recovery, they were trained using standard operant conditioning procedures to fixate visual targets for fluid reward, and to report the direction of surface slant using eye movement responses to targets located above and below the stimulus. After training, neuronal recordings began. We recorded from CIP in two monkeys (N and P), and from V3A in two monkeys (Z and P). Before the study, monkey Z underwent a bilateral labyrinthectomy as part of another project. Results from V3A in monkeys Z and P were compared statistically using Wilcoxon rank-sum tests, and no significant differences were found, indicating that the labyrinthectomy had no detectable effects on the current study. Specifically, there were no significant differences in median choice probability (CP; monkey Z, CP = 0.50; monkey P, CP = 0.47; *p* = 0.73), neuronal threshold (monkey Z, 31.29°; monkey P, 23.73°; *p* = 0.60), surface orientation discrimination index (SODI; monkey Z, SODI = 0.68; monkey P, SODI = 0.71; *p* = 0.89), squared choice partial correlation (CPC; monkey Z, *r* ^2^ = 0.003; monkey P, *r* ^2^ = 0.01; *p* = 0.20), and squared slant partial correlation (SPC; monkey Z, *r* ^2^ = 0.02; monkey P, *r* ^2^ = 0.02; *p* = 0.59). A lack of effects of the labyrinthectomy on visual discrimination is not surprising given that the monkeys were head-fixed during the experiments and that previous studies found that visual heading discrimination performance is largely normal within days following a bilateral labyrinthectomy (Gu et al., 2007).

### Data acquisition

Epoxy-coated tungsten microelectrodes (diameter, 125 µm; impedance, 1-5 MΩ at 1kHz; FHC) were inserted into the cortex through a transdural guide tube using a hydraulic microdrive to record extracellular action potentials. Neuronal voltage signals were amplified, filtered (1 Hz to 10 kHz), and displayed on an oscilloscope to isolate single units using a window discriminator (BAK Electronics). Raw voltage signals were digitized at a rate of 25 kHz using a Power 1401 data acquisition system (Cambridge Electronic Design), and single units were sorted off-line as needed (Spike2, Cambridge Electronic Design). In some experiments, action potentials were displayed and isolated using the SortClient software (Plexon).

The CARET software was used to segment visual areas in MRI scans of monkeys N and P (Lewis and Van Essen, 2000). Two MRI scans were performed with each of the monkeys. The first (baseline) scan was performed before the head restraint ring was implanted. The second scan was performed after placement of the recording grid to align the grid to the baseline MRI images. Recording sites were localized to CIP (which the Lewis and Van Essen Atlas designates as the lateral occipitoparietal zone) using the resulting MRI atlases after alignment of the grids (Van Essen et al., 2001; Rosenberg et al., 2013). When lowering an electrode dorsal-ventrally, CIP was preceded by either the intraparietal sulcus or by cells with prevalent eye-movement responses, depending on the medial-lateral position of the penetration. Once either the intraparietal sulcus or eye-movement responsive cells were passed, neurons were tested for surface orientation selectivity. Neurons in CIP were further identified as having large receptive fields often extending into the ipsilateral visual hemifield (Taira et al., 2000). Area V3A was targeted using the MRI atlas in monkey P and using stereotaxic coordinates in monkey Z. Area V3A is located ventral-lateral and adjacent to CIP. Lateral to CIP and dorsal to V3A is a large patch of white matter. Thus, both CIP and gray/white matter transitions provided landmarks for targeting V3A. As electrodes were advanced dorsal-ventrally, observed gray/white matter transitions were compared with coronal sections to localize V3A. Receptive field mapping was used to compare the receptive field sizes of V3A neurons to previously published data. Receptive field size increased with eccentricity (*r* = 0.621, *p* = 0.002), and the linear fit *y* = 0.47*x* + 1.8 was similar to previous measurements, *y* = 0.33*x* + 1.78 (Galletti and Battaglini, 1989) and *y* = 0.38*x* + 2.8 (Nakamura and Colby, 2000), as obtained using DataThief (Tummers, 2006). We compared response latency between the areas, and found that V3A neurons (median, 56 ms) responded significantly faster than CIP neurons (median, 72 ms; Wilcoxon rank-sum test, *p* = 0.02).

### Behavioral control and stimulus presentation

Behavioral control was conducted with custom Spike2 scripts. The monkeys sat in a primate chair ∼32.5 cm from a liquid crystal display (LCD) on which stimuli were displayed (System 1: Accusync LCD 93VX, NEC; System 2: 1707 FP, Dell). An aperture constructed from a black nonreflective material was affixed to the screen such that the monkey could only see stimuli within a 30-cm-diameter (System 1) or 18-cm-diameter (System 2) circular aperture. The same material extended between the LCD and the monkey, occluding the view of the surrounding room. The OpenGL graphics library was used to program visual stimuli that were generated using an OpenGL accelerator board (Quadro FX 3000G, PNY Technologies). The fixation point (yellow in color) was presented directly in front of the monkey at eye level and screen distance. Fixation was enforced using 2° vergence and 1° version windows. Due to eye coil failures in monkey P, the binocular eye movements of this animal were monitored in all experiments using an infrared optical eye tracker (ISCAN).

### 3D surface orientation tuning

**Figure 1.**
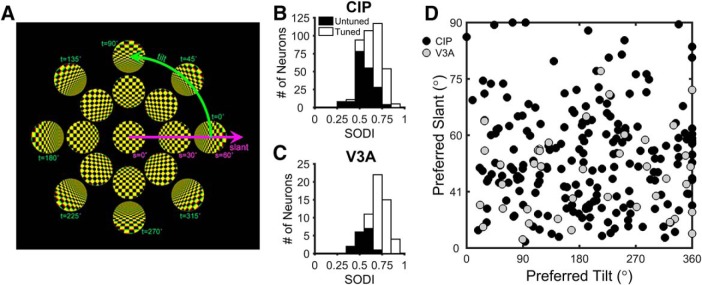
Surface orientation tuning. ***A***, The 3D orientation of a planar surface can be described by two variables, slant and tilt. Tilt specifies the axis within the frontoparallel plane about which the plane is rotated, and slant specifies how much it is rotated. These variables define a polar coordinate system. Only a subset of the stimuli used in the study are shown. ***B***, ***C***, Distributions of the SODI for 396 CIP (***B***) and 60 V3A (***C***) neurons. Open bars denote tuned neurons (215 CIP and 44 V3A), and filled bars denote untuned neurons (181 CIP and 16 V3A). ***D***, Equal area projection (Rosenberg et al., 2013) showing joint distribution of preferred slants and tilts for the 215 tuned CIP neurons (black circles) and 44 tuned V3A neurons (gray circles).

Surface orientation tuning was measured as previously described (Rosenberg et al., 2013; Rosenberg and Angelaki, 2014a,b). Briefly, a planar surface with a checkerboard pattern was used to measure the joint tuning for slant and tilt (Fig. 1*A*). Stimuli subtended either 50° or 31° of visual angle. Initial recordings with monkey N were conducted in System 1 (used in our previous CIP studies), which allowed us to present 50° stimuli (30 neurons). However, monkey N outgrew the system, which only accommodates relatively small animals. The remaining data for monkey N (14 neurons) and all data from monkeys P and Z were gathered in System 2, in which the largest possible stimulus was 31°. Wilcoxon rank-sum tests revealed no significant differences in the results for monkey N across the two systems, including the following comparisons of median values: choice probability (System 1, CP = 0.57; System 2, CP = 0.58; *p* = 0.44); neuronal threshold (System 1, 37.96°; System 2, 31.79°; *p* = 0.89); behavioral threshold (System 1, 3.60°; System 2; 3.74°; *p* = 0.27); point of subjective equality (P.S.E.; System 1, 0.16°; System 2, −0.71°; *p* = 0.13); squared choice partial correlation (System 1, *r* ^2^ = 0.02; System 2, *r* ^2^ = 0.009; *p* = 0.47); and squared slant partial correlation (System 1, *r* ^2^ = 0.01; System 2, *r* ^2^ = 0.02; *p* = 0.97).

Slant was varied between 0° and 60° in 20° steps, and tilt was varied between 0° and 315° in 45° steps. All stimuli were centered on the fixation point and covered the same retinotopic area. Stereoscopic cues were created by rendering the stimuli as red-green anaglyphs. Each trial began with the monkey fixating a point on a blank screen for 300 ms. Fixation was maintained while a checkerboard stimulus was presented for 1000 ms, followed by 50 ms of fixation with a blank screen. There was a 1000 ms blank screen intertrial interval. Stimuli were presented in pseudo-random order. Surface orientation selectivity was assessed for all cells held for at least three repetitions of each stimulus. At most, seven repetitions of each stimulus were recorded. For each selective neuron (see Results), a one-way ANOVA was performed to determine whether there was significant slant tuning along the 90°/270° tilt axis (Fig. 1*A*, see Fig. 3*A*,*B*). Neurons with significant tuning were studied further in the slant discrimination task.

### Slant discrimination task

**Figure 2.**
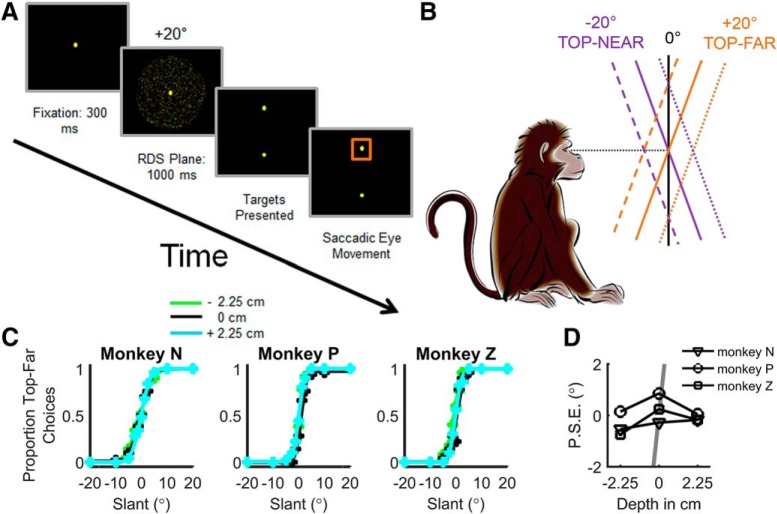
Slant discrimination task and behavioral performance. ***A***, Temporal sequence of events in the slant discrimination task. Each trial began with the presentation of a fixation point at the center of the screen. The monkey fixated this point for 300 ms after which an RDS plane was presented for 1000 ms while fixation was maintained. The monkey then reported which direction the plane was slanted away from frontoparallel by making a saccade to the upper target if the slant was positive (top-far) or the lower target if the slant was negative (top-near). ***B***, Side view of the task illustrating positive versus negative slants. Solid lines depict planes centered at the fixation depth (screen distance, ∼32.5 cm). Dashed and dotted lines depict planes centered at either near or far depths (2.25 cm in front of or behind the display), respectively. ***C***, Discrimination behavior plotted as the proportion of top-far choices as a function of slant. Data are fit with a cumulative Gaussian for each depth (*N* = 450 trials/data point). ***D***, The P.S.E. as a function of depth for each monkey. For comparison, the gray line shows the expected dependency of the P.S.E. on stimulus depth if the task was performed based on local absolute disparities rather than slant. The line reaches ±14° at ±2.25 cm but is clipped at ±2° to not obscure the data.

The slant discrimination task was always performed along the 90°/270° tilt axis. To simplify the description of surface orientation, we do not refer to tilt for the slant discrimination task but instead denote planes with a tilt of 90° (top of the plane closer to the monkey) as having a negative slant, and planes with a tilt of 270° (top of the plane further from the monkey) as having a positive slant (Rosenberg and Angelaki, 2014b). As illustrated in Figure 2*A*, each trial of the slant discrimination task began with the monkey fixating a target on a blank screen for 300 ms, after which a random dot stereogram (RDS) depicting a planar surface was presented for 1000 ms. After presentation of the RDS, the fixation point disappeared, and two choice targets appeared 8.6° above/below the location of the fixation point. The monkey then made an eye movement to one of the choice targets to indicate the perceived slant. Correct responses were defined as a saccade to the upper target when the slant was positive (top-far) or to the lower target when the slant was negative (top-near). Correct responses were rewarded with a drop of water or juice. For planes with slant = 0° (i.e., frontoparallel), responses were rewarded pseudo-randomly 50% of the time. If the monkey broke fixation at any point during the stimulus presentation, the trial was aborted and the data discarded.

During pilot work, we observed that local orientation cues in checkerboard stimuli could be used to perform the task without having to judge slant. To avoid this potential confound, the discrimination task was performed using RDS planes with uniform dot density on the screen (Sanada et al., 2012). In CIP, slant tuning curves measured with planar surfaces with a checkerboard pattern or a random dot pattern are highly correlated (Rosenberg and Angelaki, 2014b). To discourage the monkeys from using local depth cues to perform the task (Hillis et al., 2004), we varied the mean depth (near = −2.25 cm from the screen; screen distance = 0 cm; far = 2.25 cm from the screen) of the RDS plane from trial to trial (Fig. 2*B*). This discouraged them from judging whether the upper (lower) half of the stimulus was in front of (behind) the plane of the display. If the animals relied on the absolute disparity of a subregion of the stimulus to perform the task, large behavioral biases would result at the near/far depths. For the 31° stimulus, biases of at least 14° in magnitude (the slant at which a stimulus would start to cross the screen) would occur in opposite directions for the near and far depths. Behavioral data clearly show that this was not the case (Fig. 2*C*,*D*), suggesting that the animals correctly learned to judge the sign of slant. To maintain this behavior during the neuronal recordings, stimuli were presented at screen distance for 70% of trials, the near depth for 15% of trials, and the far depth for 15%. For the neuronal recordings, there were sufficient data to reliably analyze the responses measured at screen distance only.

Slant was varied between ±20° with the intermediate slants tailored to each monkey’s performance. For monkeys N and Z, slants of ±20°, 10°, 5°, 2.5°, 1.25°, and 0° were used. For monkey P, slants of ±20°, 9°, 4.05°, 1.83°, 0.82°, and 0° were used. Neurons were recorded while the monkey performed the task for a minimum of 10 repetitions of each stimulus. Sufficient repetitions were recorded for 65 CIP and 23 V3A neurons.

### Data analysis

Analyses were performed in MATLAB (MathWorks). Unless otherwise noted, analyses were performed on firing rates or spike counts computed during the 1000 ms stimulus presentation period. The tuning strength of each neuron was evaluated using a SODI, motivated by previous studies (Prince et al., 2002), that was calculated using the full slant–tilt tuning curve. The SODI quantifies the strength of response modulation relative to overall response variability as follows:

(1) $$SODI=\frac{R_{max}-R_{min}}{R_{max}-R_{min}+2\sqrt{SSE/(N-M)}},$$

where *R*_max_ and *R*_min_ are the maximum and minimum responses, respectively. SSE denotes the sum squared error around the mean responses, *N* is the total number of trials, and *M* is the number of tested slant–tilt combinations (*M* = 25). Neurons with strong response modulation relative to their variability have SODI values closer to 1, whereas neurons with weak response modulation have SODI values closer to 0.

Behavioral performance in the slant discrimination task was quantified by plotting the proportion of top-far choices as a function of stimulus slant. The resulting psychometric function was fit with a cumulative Gaussian using the Psignifit toolbox (Wichmann and Hill, 2001). The point of subjective equality and behavioral threshold were defined as the mean and SD of the cumulative Gaussian fit, respectively.

Neuronal sensitivity was measured by using ROC analysis to assess the ability of an ideal observer to discriminate non-zero from zero slants (e.g., −20° from 0°) (Britten et al., 1996; Gu et al., 2007). To construct a neurometric function that could be directly compared with the psychometric function (Fig. 4*A*,*B*), ROC values were plotted as a function of slant and fit with a cumulative Gaussian using the Psignifit toolbox. Neuronal threshold (an inverse measure of sensitivity) was defined as the SD of the cumulative Gaussian fit. Neuronal and behavioral thresholds were calculated from simultaneously gathered data, allowing for a direct comparison. For this comparison, neuronal thresholds were multiplied by $\sqrt{2}$ to account for the behavioral task being conducted as a one-interval task (Hillis et al., 2004), but the neurometric functions being calculated by comparing zero and non-zero response distributions. The time course of neuronal sensitivity was assessed by computing neuronal thresholds in 200 ms time windows, starting at 100 ms after stimulus onset, and shifted every 50 ms over the 1000 ms stimulus duration.

**Figure 3.**
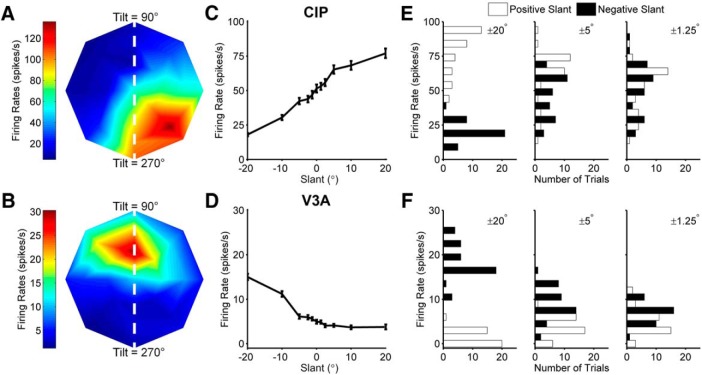
Surface orientation tuning of example CIP and V3A neurons. ***A***, ***B***, Slant–tilt tuning profiles of representative CIP (***A***) and V3A (***B***) neurons. Firing rate is color coded with red hues indicating larger firing rates. The peak of the CIP tuning profile is in the lower right corner, indicating that the cell responded best to a planar surface with the lower right corner closest to the monkey. The peak of the V3A tuning profile is in the upper portion of the plot, indicating that the cell responded best to a planar surface with the top closest to the monkey. White dashed lines correspond to the 90°/270° tilt axis along which the slant discrimination task was performed. ***C***, ***D***, Slant tuning curves of the same CIP (***C***) and V3A (***D***) neurons measured during the slant discrimination task. Error bars denote SEM. ***E***, ***F***, Neuronal response distributions for three pairs of slant angles (±20°, ±5°, ±1.25°) for the CIP (***E***) and V3A (***F***) neurons. Negative slants are shown as black bars and positive slants are shown as white bars.

**Figure 4.**
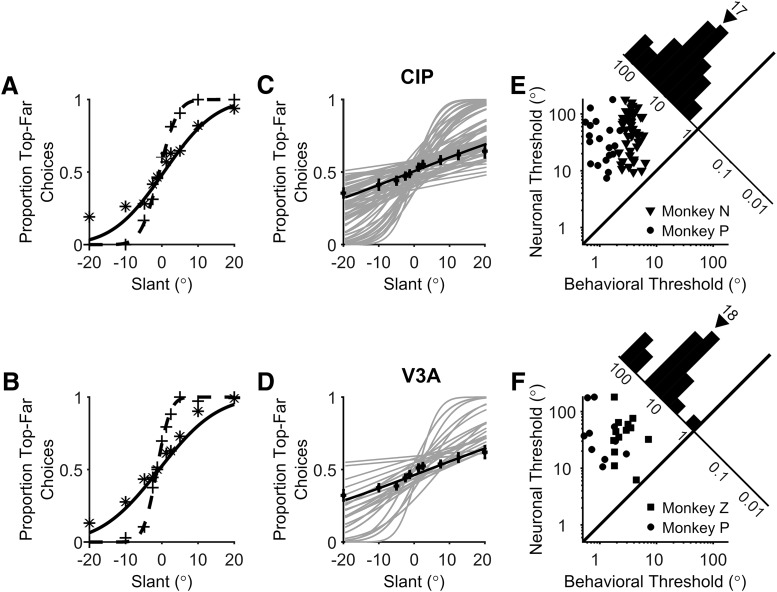
Comparison of behavioral and neuronal sensitivity. ***A***, ***B***, The proportion of top-far choices made during the recordings of the CIP (***A***) and V3A (***B***) neurons from Figure 3 are plotted as a function of slant (+ symbols). Simultaneously measured neuronal responses were converted into neurometric functions using ROC analysis and the proportion of top-far choices of an ideal observer are plotted as a function of slant (* symbols). Dashed and solid curves show cumulative Gaussian fits to the psychometric and neurometric functions, respectively. ***C***, ***D***, Gray curves show cumulative Gaussian fits to the neurometric functions of each neuron recorded during the slant discrimination task. Black symbols and curves show average neurometric functions across animals and neurons. Error bars denote SEM. ***E***, ***F***, Behavioral and neuronal thresholds are compared for all individual experiments for monkeys N (triangles), P (circles), and Z (squares) for CIP (***E***) and V3A (***F***). Neuronal thresholds are multiplied by $\sqrt{2}$. Diagonal histograms show distributions of neuronal to behavioral threshold ratios. Triangles above the histograms mark median threshold ratios.

To quantify the relationship between neuronal response and choice, CPs were computed using ROC analysis. For each slant, neuronal responses were grouped according to the choice. “Preferred” choices corresponded to those made in favor of the preferred slant of the neuron, as determined from the 3D surface orientation tuning profile measured during fixation. “Nonpreferred” choices corresponded to those made in the opposite direction. CP was computed by performing ROC analysis on the preferred and nonpreferred choice distributions for the (ambiguous) 0° slant stimulus. To achieve greater statistical power, a grand CP was computed by performing ROC analysis after normalizing the neuronal responses for each stimulus slant and combining the normalized data into two composite distributions corresponding to preferred versus nonpreferred choices (Kang and Maunsell, 2012). Only stimulus slants for which the monkey made at least three choices in each direction were included in the grand CP calculation. To test whether CPs were significantly different from chance level (CP = 0.50), a permutation test was used (1000 permutations). The time course of choice-related activity was measured by computing CPs in 200 ms time windows, starting at 100 ms after stimulus onset, and shifted every 50 ms. The last time window was centered 150 ms after the stimulus offset (1150 ms after stimulus onset). In this way, the time course of choice-related activity included responses up to approximately the median choice time (271 ms after stimulus offset).

To quantify the contributions of stimulus slant and choice to the responses of each neuron, Pearson correlations were computed between the following variables: slant (S), choice (C), and neuronal spike count (F). From these correlations, we computed a slant partial correlation, *r*_FS.C_ (Eq. 2) that quantifies the relationship between F and S while controlling for C, and a choice partial correlation, *r*_FC.S_ (Eq. 3), that quantifies the relationship between F and C while controlling for S. Because this analysis assumes a linear relationship between the stimulus and firing rate over the range of tested slants, we confirmed that the pattern of results did not change if slant was replaced with a nonlinear slant function including cubic, exponential, and sigmoidal functions, or if a larger partial correlation analysis was run that included multiple slant functions including the linear term. We did not consider nonlinear functions of choice because choice was a binary variable. Because the pattern of results did not depend appreciably on the stimulus function, as also reported recently for heading discrimination in the ventral intraparietal area (Zaidel et al., 2017), only the partial correlation analysis performed with slant, choice, and spike count is presented.

(2) $$r_{FS.C}=\frac{r_{FS}-r_{FC}r_{SC}}{\sqrt{\left(1-r_{FC}^{2})(1-r_{SC}^{2}\right)}}$$

(3) $$r_{FC.S}=\frac{r_{FC}-r_{FS}r_{SC}}{\sqrt{\left(1-r_{FS}^{2})(1-r_{SC}^{2}\right)}}.$$

Positive slant partial correlations indicate that spike counts were greater for positive slants than negative slants. Positive choice partial correlations indicate that spike counts were greater for top-far than top-near choices. Partial correlations were computed based on spike counts over the entire 1000 ms stimulus duration. For the time course analyses, partial correlations were computed in 200 ms time windows, starting at 100 ms after stimulus onset, and shifted every 50 ms. The last bin center was 1150 ms after stimulus onset. For the partial correlation time course analysis, partial correlations were squared to determine how much variance in the spike counts was accounted for by stimulus and choice.

## Results

### Comparison of CIP and V3A responses to 3D surface orientation

Surface orientation tuning was measured for 427 CIP and 72 V3A neurons during a fixation task in which a checkerboard plane was presented at 25 slant–tilt combinations (Fig. 1*A*). Of these, 396 CIP (93%) and 60 V3A (83%) neurons were held for enough repetitions (three or more) to assess tuning. Tuning strength was quantified using a SODI (see Materials and Methods), which ranges from 0 to 1. Larger SODI values indicate stronger tuning. The mean SODI in CIP was 0.63 ± 0.005 SEM (*N* = 396; Fig. 1*B*), and in V3A it was 0.68 ± 0.02 SEM (*N* = 60; Fig. 1*C*). The mean SODI was significantly smaller in CIP than V3A (Wilcoxon rank-sum test, *p* = 5.8 × 10^−4^).

A two-step procedure was used to classify neurons as tuned or untuned. First, a one-way ANOVA was performed on the firing rates in response to each of the 25 slant–tilt combinations. Second, the tuning curve of each neuron that passed the ANOVA (*p* < 0.05) was fit with a Bingham function (Rosenberg et al., 2013). The second step eliminates neurons with multiple tuning peaks that would pass an ANOVA but are not selective for a unique stimulus (Rosenberg et al., 2013, their Fig. 5). Neurons with a Pearson correlation for the Bingham fit ≥0.8 were classified as tuned, and otherwise untuned. Based on these criteria, 215 CIP neurons (54% of the 396 tested) and 44 V3A neurons (73% of the 60 tested) were tuned. Of the neurons classified as untuned, 26.5% in CIP (48 of 181) and 25% in V3A (4 of 16) were rejected for having multiple peaks.

**Figure 5.**
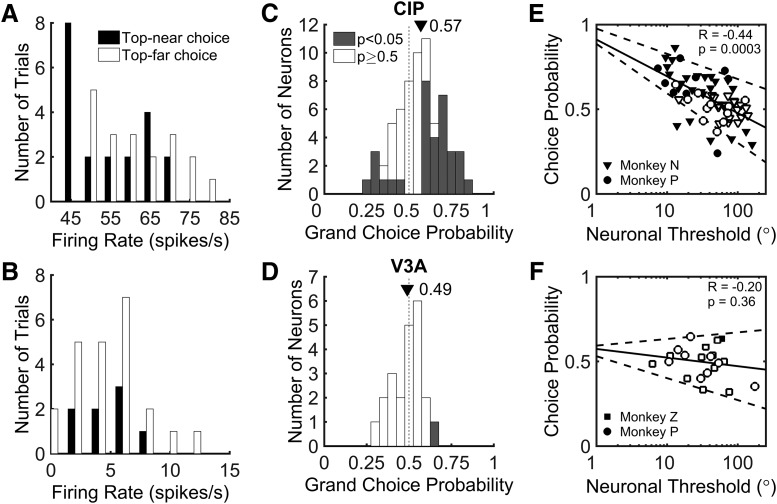
Summary of choice-related activity in CIP and V3A. ***A***, ***B***, Distribution of firing rates for example CIP (***A***) and V3A (***B***) neurons (same as in Figs. 3, 4*A*,*B*) in response to the ambiguous 0° slant stimulus. Responses are sorted according to whether the monkey made a top-near (black) or top-far (white) choice. For the CIP neuron, the choice-related difference in responses yielded a choice probability significantly different from chance (grand CP = 0.65, *p* = 0.001). For the V3A neuron, there was no significant choice-related difference in responses (grand CP = 0.50, *p* = 0.36). ***C***, ***D***, Histograms of grand choice probabilities for all 65 CIP (***C***) and 23 V3A (***D***) neurons. Gray bars denote CPs that are significantly different from the chance value of 0.50 (*p* < 0.05, permutation test). Mean CPs are marked by triangles. ***E***, ***F***, Choice probability as a function of neuronal threshold (multiplied by $\sqrt{2}$). There is a significant negative correlation between CP and neuronal threshold in CIP (***E***) and no significant correlation between CP and neuronal threshold in V3A (***F***). Solid lines show linear fits and dashed lines show 95% confidence intervals for the slope. Filled symbols denote CPs significantly different from chance (0.50, *p* < 0.05, permutation test). Different symbols correspond to different animals.

The distribution of slant–tilt preferences was examined for each area by performing an equal area preserving projection (Rosenberg et al., 2013) and plotting the preferred slant and tilt of each neuron in that space (Fig. 1*D*). We previously found that the distribution of CIP slant–tilt preferences was not significantly different from uniform in untrained animals (Rosenberg et al., 2013). Here we found that the distribution of preferences in CIP and V3A were significantly different from uniform (χ^2^ test: CIP, *p* = 1.07 × 10^−7^; V3A, *p* = 0.01). In particular, there was a bias toward representing smaller slants (note the relative sparsity of cells near the top of the scatter plot in Fig. 1*D*). It is possible that extensive training in the fine slant discrimination task resulted in a shift in tuning preferences toward smaller slants.

### Slant discrimination behavior

A control experiment was conducted to confirm that the animals did not perform the slant discrimination task based on local absolute disparity cues signaling that the upper (lower) half of the plane was in front of (behind) the LCD. Each monkey performed the slant discrimination task for nine sessions with the stimuli centered at three depths (0 and ±2.25 cm) from the display (Fig. 2*A*,*B*). Psychometric functions for each monkey and depth are shown in Figure 2*C*. The proportion of “top-far” choices is plotted for each slant and fit with a cumulative Gaussian function. One-way ANOVAs showed no significant effect of depth on the P.S.E. (monkey N, *F* = 0.65, *p* = 0.53; monkey P, *F* = 0.53, *p* = 0.60; monkey Z, *F* = 2.41, *p* = 0.12) or threshold (monkey N, *F* = 0.58, *p* = 0.57; monkey P, *F* = 0.11, *p* = 0.90; monkey Z, *F* = 0.70, *p* = 0.51). Although not significant, there was a slight tendency for the P.S.E. to be negative at −2.25 cm (Fig. 2*D*). However, if the animals were relying on local absolute disparity cues to perform the task, the P.S.E. would have a magnitude of at least 14° at the near/far depths (i.e., the smallest slant at which a plane would cross the screen), which is much greater than the average P.S.E. of −0.38° at −2.25 cm. One-way ANOVAs also revealed that there was no significant effect of stimulus depth on mean vergence angle during the stimulus presentation (monkey N, *F =* 0.42, *p =* 0.70; monkey P, *F =* 3.57 × 10^−4^, *p* = 0.99; monkey Z, *F* = 0.50, *p* = 0.66), suggesting that the slightly negative P.S.E. at −2.25 cm was not due to a systematic vergence error. These data strongly suggest that the monkeys performed the task by assessing the slant of the plane rather than by judging local stimulus depth relative to the plane of fixation.

### Neuronal sensitivity during slant discrimination

Of the 215 tuned CIP neurons, 151 (70%) were significantly tuned for slant (ANOVA, *p* < 0.05) along the 90°/270° tilt axis used in the slant discrimination task (Fig. 3*A*,*B*, white dashed lines) and therefore was studied further. Of these, data from 65 CIP neurons (43%) were included in this study. The remaining 86 neurons (57%) were recorded for another task (16 neurons, 11%) or were not recorded for a sufficient number of repetitions (≥10) to be included (70 neurons, 46%). Likewise, of the 44 V3A neurons, 35 (80%) were significantly tuned for slant along the 90°/270° tilt axis. Of these, 23 (66%) were held for sufficient repetitions (≥10) to be included.

Surface orientation tuning curves of example CIP and V3A neurons that met these criteria are shown in Figure 3, *A* and *B*. Responses recorded during the slant discrimination task are shown in Figure 3, *C* and *D* for the same neurons. For both neurons, tuning was monotonic over the range of slants presented in the discrimination task. The CIP neuron (Fig. 3*C*) fired more in response to positive slants (top of the plane further from the animal), whereas the V3A neuron (Fig. 3*D*) fired more in response to negative slants (top of the plane closer to the animal).

Firing rate distributions for three pairs of slants (±20°, ±5°, and ±1.25°) are shown in Figure 3, *E* and *F*. To assess how well the responses of the neurons could be used to discriminate non-zero from zero slants, we compared the firing rate distribution for each non-zero slant to the firing rate distribution for the frontoparallel (slant = 0°) plane. The ability of an ideal observer to discriminate non-zero slants from the frontoparallel plane was quantified using ROC analysis (Britten et al., 1996; Gu et al., 2007). The probability that an ideal observer would report that the slant of a presented plane was positive was calculated for each non-zero slant. A neurometric function was then constructed by plotting the ROC value for each slant and fitting the function with a cumulative Gaussian (Fig. 4*A*,*B*, solid curves). A neuronal threshold quantifying the sensitivity of the neuron to changes in slant was defined as the SD of the cumulative Gaussian fit. This analysis was performed for each of the 65 CIP and 23 V3A neurons, and the resulting neurometric functions are shown in Figure 4, *C* and *D*. Across all monkeys, the median neuronal thresholds were 32.86° in CIP and 26.25° in V3A, and were not significantly different (Wilcoxon rank-sum test, *p* = 0.48). We further confirmed that neuronal thresholds were similar between monkeys. The median CIP thresholds were 35.16° (monkey N) and 26.04° (monkey P), and not significantly different (Wilcoxon rank-sum test, *p* = 0.30). Likewise, the median V3A thresholds were 31.30° (monkey Z) and 23.73° (monkey P), and were not significantly different (Wilcoxon rank-sum test, *p* = 0.58). These results indicate that CIP and V3A neurons are similarly sensitive to changes in slant.

Neurometric functions can be directly compared with psychometric functions measured in the same recording session (Fig. 4*A*,*B*). Simultaneously measured neuronal and behavioral thresholds are compared in Figure 4, *E* and *F*, for CIP and V3A, respectively. For this comparison, neurometric thresholds were multiplied by $\sqrt{2}$ since the neurometric functions were constructed by comparing two response distributions, whereas the behavioral task had a single stimulus interval. Distributions of neuronal-to-behavioral threshold ratios are shown as diagonal histograms. All of the neuronal/behavioral threshold ratios were >1, indicating that no recorded CIP or V3A neuron was more sensitive than the monkey. The median neuronal/behavioral threshold ratio of monkey N was 14 for CIP, the median threshold ratio of monkey P was 34 for CIP and 30 for V3A, and the median threshold ratio of monkey Z was 16 for V3A. Although behavioral sensitivity was greater than neuronal sensitivity, the thresholds of some neurons approached that of the behavior, suggesting that CIP and V3A could contribute to performance of the slant discrimination task.

### Neuronal responses in CIP but not V3A correlated with slant reports

During the slant discrimination task, variability was observed in both the neuronal firing rates and choices elicited by stimuli of the same slant. This variability is evident in histograms of the responses of the example CIP neuron to a slant of 0°, grouped by choice (Fig. 5*A*). This stimulus is ambiguous, and there is no correct answer because the top of the plane leans neither toward nor away from the monkey. Thus, the monkey chose each response target with approximately equal frequency. For the example CIP neuron, the firing rate tended to be lower when the monkey made a top-near choice and greater when the monkey made a top-far choice. In other words, responses were greater when the monkey chose the target corresponding to the slant preference of the neuron. In contrast, the example V3A neuron preferred negative slants, but the histograms of responses to a slant of 0°, grouped by choice, were largely overlapping. Thus, there was no clear difference in the activity of the example V3A neuron when the animal made top-far versus top-near choices (Fig. 5*B*).

CP analysis was used to quantify the relationship between neuronal response and choice (Celebrini and Newsome, 1994; Britten et al., 1996; Dodd et al., 2001; Nienborg and Cumming, 2006; Gu et al., 2007). We computed the CP by first assigning neuronal responses, calculated over the 1000 ms stimulus presentation period, to two groups according to the monkey’s choice. Preferred slant choices were made in the direction of the preferred slant and nonpreferred slant choices were made in the direction of the nonpreferred slant. Preferred and nonpreferred slants were defined according to the tuning preference along the 90°/270° tilt axis that was measured during the 3D orientation tuning (fixation only) task. Slant preferences generally matched between the fixation and discrimination tasks, with the preference reversing for only six CIP neurons and one V3A neuron. Since reversals of slant preference could be an effect of choice-related signals during the discrimination task, we computed CPs based on stimulus preferences measured during fixation.

After sorting responses by choice, we used ROC analysis to compute the probability that an ideal observer could predict the choice of the monkey based on the responses of the neuron (see Materials and Methods). The CP was calculated in two ways. First, we only considered responses to the ambiguous 0° slant stimulus. For the CIP neuron in Figure 5*A*, the CP was 0.65, indicating it fired more when the monkey made a choice in favor of the preferred slant. Across all CIP neurons, the mean CP for a 0° slant stimulus was 0.58, which was significantly greater than the chance value of 0.50 (*t* test: *t* = 3.89, *p* = 2.45 × 10^−4^). For the V3A neuron in Figure 5*B*, the CP was 0.45, suggesting that the neuron fired slightly more when the monkey made a choice in favor of the nonpreferred slant of the cell. Across all V3A neurons, the mean CP for the 0° slant stimulus was 0.52, which was not significantly different from chance (*t* test: *t* = 0.64, *p* = 0.53). Second, to achieve greater statistical power, we calculated a “grand CP” by including responses to all slants for which the monkey made at least three choices toward each response target. For this analysis, responses to each slant were normalized using the balanced *z*-score method (Kang and Maunsell, 2012). For the CIP neuron in Figure 5*A*, the grand CP was 0.65 and significantly greater than the chance value of 0.50 (permutation test, 1000 permutations, *p* = 0.001). The grand CP for the V3A neuron in Figure 5*B* was 0.50 and was not significantly different from chance (*p* = 0.36). Across the neural populations, the grand CP was highly correlated with the CP measured for the 0° slant stimulus (CIP, *r* = 0.81, *p* = 1.0 × 10^−15^; V3A, *r =* 0.78, *p* = 0.0001). The analyses that follow are based on grand CPs.

Histograms of grand CP for CIP and V3A are shown in Figure 5, *C* and *D*. The mean CIP CP was 0.57, which was significantly >0.50 (*t* test, *p* = 1 × 10^−15^). The mean CIP CP was also significantly different from chance for each monkey (*t* test: monkey N, CP = 0.57, *p* = 3.40 × 10^−4^; monkey P, CP = 0.57, *p* = 0.04). In total, 51% of CIP neurons (33 of 65) had CPs that were significantly different from chance (permutation test, 1000 permutations, *p* < 0.05). For the majority of CIP neurons with significant CPs (26 of 33), firing rates increased when the monkey made a choice in favor of the preferred slant (CPs > 0.50). However, 7 CIP CPs were significantly <0.50, indicating that they fired more when the monkey made a choice in favor of the nonpreferred slant. In contrast to CIP, the mean V3A CP was 0.49, which was not significantly different from 0.50 (*t* test, *p* = 0.40). Neither monkey had a mean V3A CP that was significantly different from chance (*t* test: monkey P, CP = 0.48, *p* = 0.42; monkey Z, CP = 0.49, *p* = 0.67). Permutation tests revealed that only one V3A neuron had a CP that was significantly different from chance. As a control, we confirmed that there was no significant difference in CP associated with whether the neurons preferred positive or negative slants. The mean CIP CP was 0.55 ± 0.03 SEM (*N* = 30) for neurons preferring positive slants and 0.59 ± 0.03 SEM (*N* = 35) for those preferring negative slants (*t* test, *t* = 1.49, *p* = 0.14). The mean V3A CP was 0.46 ± 0.03 SEM (*N* = 10) for neurons preferring positive slants and 0.50 ± 0.03 SEM (*N* = 13) for those preferring negative slants (*t* test, *t* = 1.29, *p* = 0.21). Comparing choice-related activity across the two areas, we found that the mean CIP CP was significantly greater than the mean V3A CP (*t* test, *p* = 0.003). These findings indicate that the CIP, but not V3A, neurons displayed strong choice-related activity during the slant discrimination task.

We further found that the CIP neurons showed a significant negative correlation between neuronal threshold and CP (*r* = −0.44, *p* = 3 × 10^−4^; Fig. 5*E*). The 10 most sensitive CIP neurons had a mean CP of 0.72 ± 0.03 (SEM), whereas the 10 least sensitive had a mean CP of 0.48 ± 0.03 (SEM). In contrast, the correlation between neuronal threshold and CP was not significant in V3A (*r* = −0.20, *p* = 0.36), and the V3A CPs clustered around 0.50 regardless of neuronal threshold (Fig. 5*F*). We additionally ran an analysis of covariance (ANCOVA) in which CP was the dependent variable, neuronal threshold was a continuous covariate, and brain area was an ordinal factor. We found a significant interaction (*p* = 0.03) between neuronal threshold and brain area, indicating a significant difference in the strength of the relationship between CP and neuronal threshold in CIP and V3A.

As a control, we confirmed that trial-by-trial variation in vertical eye position, vertical eye velocity, and vergence during the stimulus presentation had no appreciable effect on CIP CPs and neuronal thresholds (Gu et al., 2007). For each CIP neuron, we performed three separate ANCOVAs to test the relationship between neuronal firing rate and choice with vertical eye position, vertical eye velocity, or vergence as coregressors (averaged over the length of each trial). Fifteen percent of CIP neurons (10 of 65) had a significant dependence of firing rate on vertical eye position, 3% (2 of 65) had a significant dependence of firing rate on vertical eye velocity, and 6% (4 of 65) had a significant dependence of firing rate on vergence (*p* < 0.05, ANCOVA, Bonferroni–Holm correction for multiple comparisons). We therefore calculated CPs and neuronal thresholds after removing the dependence (linear trend) on vertical eye position, vertical eye velocity, and vergence from the neuronal responses. After removing the effect of vertical eye position, there was a small but significant reduction in CP (0.57 before vs 0.56 after correction; paired *t* test, *t* = 2.53, *p* = 0.01). The CP measurements before and after correction were highly correlated (*r* = 0.96, *p* = 1.0 × 10^−16^), and the mean value remained significantly greater than chance after correction (*t* test, *t* = 3.61, *p* = 5.95 × 10^−4^). Removal of the effect of vertical eye position had no significant effect on the median neuronal threshold (Wilcoxon sign-rank test, *p* = 0.24). For vertical eye velocity, there was a small but significant effect on the mean CP (0.57 before vs 0.56 after correction; paired *t* test, *t* = 3.05, *p* = 0.003) and the median neuronal threshold (32.86° before vs 38.06° after correction; Wilcoxon sign-rank test, *p* = 0.03). The CP measurements before and after correction were highly correlated (*r* = 0.95, *p* = 1.0 × 10^−16^), and remained significantly greater than chance after correction (*t* test, *t* = 3.57, *p* = 6.92 × 10^−4^). Neuronal thresholds were also highly correlated before and after correction (*r* = 0.85, *p* = 3.0 × 10^−15^). For vergence, there was no significant effect on mean CP (*p* = 0.58) or median neuronal threshold (*p* = 0.48). Thus, variations in eye position, eye velocity, and vergence had little effect on CIP CPs and neuronal thresholds.

### Contributions of stimulus and choice to CIP and V3A responses

**Figure 6.**
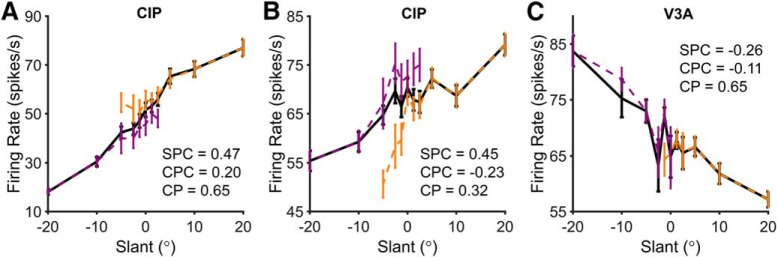
Example CIP and V3A neurons illustrating the effect of choice on slant tuning. For each neuron, the black curve shows the slant tuning curve created by averaging responses regardless of choice. Orange and purple curves show choice-conditioned slant tuning curves created by separating responses into top-far vs top-near choices, respectively. The SPC, CPC, and CP are listed for each neuron. ***A***, CIP neuron with a positive SPC, a positive CPC, and a CP > 0.50 (*p* = 0.001). ***B***, CIP neuron with a positive SPC, a negative CPC, and a CP < 0.50 (*p* = 0.001). ***C***, V3A neuron with a negative SPC, a negative CPC, and a CP > 0.50 (*p* = 0.29).

During the slant discrimination task, both the stimulus and the choice may contribute to neuronal activity. The contributions of stimulus and choice to the activity of example CIP and V3A neurons is shown in Figure 6. Slant tuning curves measured by averaging firing rates across all presentations of each slant, without regard to the choice, are shown in black. For comparison, choice-conditioned slant tuning curves were computed for top-far and top-near choices (Fig. 6, orange and purple curves, respectively). Only slants for which the monkey made at least three choices in the relevant direction were included in the choice-conditioned tuning curves. In CIP, choice-conditioned tuning curves often showed clear separation, indicating a strong effect of choice on firing rate. For the CIP neuron in Figure 6*A*, the top-far choice-conditioned tuning curve (orange) lies above the top-near choice-conditioned tuning curve (purple). This difference indicates that the neuron responded more strongly when the monkey made a choice in the direction of the preferred slant of the neuron (top-far). Correspondingly, the CP of the neuron is >0.50. In contrast, Figure 6*B* shows a CIP neuron that responded more strongly when the monkey made a choice in the opposite direction of the preferred slant. Hence, the top-near choice-conditioned tuning curve (purple) is above the top-far choice-conditioned tuning curve (orange), and the CP is <0.50. In V3A, choice-conditioned tuning curves largely overlapped. This was the case even when the CP was relatively large, as shown for the neuron in Figure 6*C*, indicating that choice had little effect on V3A responses.

To dissociate the contributions of stimulus and choice to the responses of each neuron, partial correlations were computed between slant, choice, and spike counts (over the 1000 ms stimulus presentation period) using all trials. This analysis estimates how much variance in the responses can be accounted for by stimulus and choice while controlling for the fact that these variables are correlated. Similar percentages of CIP (46%; 30 of 65) and V3A (43%; 10 of 23) neurons had significant slant partial correlations (*p* < 0.05), and the magnitude (absolute value) of the slant partial correlations in CIP (median, 0.09) and V3A (median, 0.15) were not significantly different (Wilcoxon rank-sum test, *p =* 0.14). The ranges of slant partial correlations in CIP (*r* = −0.51 to 0.47) and V3A (*r* = −0.48 to 0.46) were also similar. Correspondingly, the variance of the slant partial correlations was not significantly different between the areas (Levene’s test, W = 2.11, *p* = 0.15).

Although the slant partial correlations in CIP and V3A were similar, the choice partial correlations differed substantially. A greater percentage of neurons had significant choice partial correlations in CIP (62%; 40 of 65) than V3A (30%; 7 of 23), and the magnitude of the choice partial correlations in CIP (median, 0.13) was significantly greater than in V3A (median, 0.09; Wilcoxon rank-sum test, *p* = 0.003). The range of choice partial correlations was also greater in CIP (*r* = −0.55 to 0.49) than V3A (*r* = −0.15 to 0.20). Correspondingly, the variance of the choice partial correlations was significantly different between the areas (Levene’s test, W = 9.19, *p* = 0.003). These findings confirm that choice had a greater effect on CIP than V3A activity.

In CIP, the relative signs of the slant and choice partial correlations were largely predictive of CP. The CIP neuron in Figure 6*A* preferred positive slants (positive slant partial correlation) and top-far choices (positive choice partial correlation). Consistent with this, the CP was significantly >0.50 (*p* = 0.001). In contrast, the CIP neuron in Figure 6*B* preferred positive slants (positive slant partial correlation) but top-near choices (negative choice partial correlation). Consistent with this, the CP was significantly <0.50 (*p* = 0.001). For comparison, a V3A neuron that preferred negative slants and top-near choices is shown in Figure 6*C*. Although the CP was >0.50, it was not significantly different from 0.50 (*p* = 0.29).

**Figure 7.**
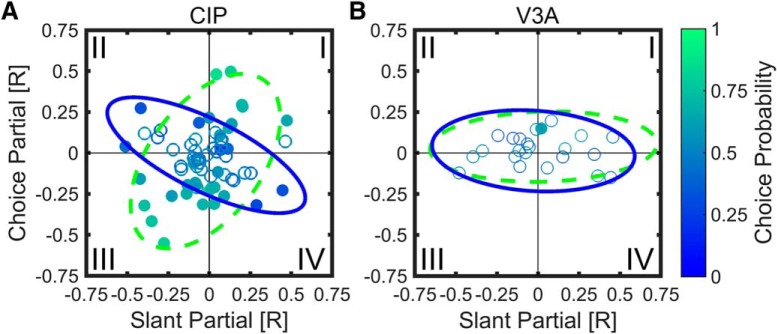
Partial correlation analysis showing relationships between slant partial correlation, choice partial correlation, and CP. Choice partial correlation is plotted as a function of slant partial correlation with individual neurons color coded to indicate CP. Significant CPs are filled, nonsignificant CPs are open. ***A***, ***B***, Data are shown for 65 CIP (***A***) and 23 V3A (***B***) neurons. Curves show 95% confidence ellipses fit to data points with CP > 0.50 (green dashed) or CP < 0.50 (blue solid). ***A***, In CIP, as indicated by the oblique orientations of the 95% confidence ellipses, CPs > 0.50 (greener) tended to occur when the slant and choice partial correlations had the same sign (quadrants I and III), whereas CPs < 0.50 (bluer) tended to occur when the slant and choice partial correlations had opposite signs (quadrants II and IV). ***B***, For V3A, choice-related activity was weak, as indicated by the elongated but horizontally oriented 95% confidence ellipses.

The relationships among slant partial correlation, choice partial correlation, and CP are summarized for CIP and V3A in Figure 7. Quadrant I (top right) contains neurons for which positive slants and top-far choices increased firing rate. Quadrant III (bottom left) contains neurons for which negative slants and top-near choices increased firing rate. Note that top-far (top-near) choices were correct for positive (negative) slants; thus, quadrants I and III contain neurons with congruent stimulus and choice effects. Based on the example cells in Figure 6, quadrants I and III should contain neurons with CPs >0.50, at least in CIP where choice effects are robust. Consistent with this prediction, the CP of 35 of 40 of the CIP neurons (88%) in quadrants I and III was >0.50 (Fig. 7*A*), and the mean CP was 0.59 ± 0.02 (SEM, *N* = 40), which was significantly >0.50 (Wilcoxon signed rank test, *p* = 3.3 × 10^−6^).

There was also a substantial number of neurons for which slant and choice had opposite effects on firing rate (quadrants II and IV). Cells in quadrant II (top left) are those for which firing rate increased for negative slants and top-far choices. Cells in quadrant IV (bottom right) are those for which firing rate increased for positive slants and top-near choices. Assuming that CP was computed based on the true sign of the slant preference (determined from the surface orientation tuning curve measured during fixation to minimize choice-related activity; the sign reversed for one CIP neuron in quadrants II/IV if determined from the slant discrimination data), neurons in quadrants II and IV should have CPs <0.50. This was not immediately evident: 12 of 25 CIP neurons (48%) in these quadrants had CPs <0.50, and the mean CP = 0.50 ± 0.02 SEM was not significantly different from 0.50 (*N* = 25, Wilcoxon signed rank test, *p* = 0.95). Note, however, that neurons with the lowest CPs (darker blue points) are largely found in quadrants II and IV.

To further test whether CPs are related to the relative signs of the slant and choice partial correlations, we fit a 95% confidence ellipse to the data from all CIP neurons with CPs >0.50 (green dashed ellipse) and a 95% confidence ellipse to those with CPs < 0.50 (blue solid ellipse), as shown in Figure 7*A*. Consistent with our predictions, the ellipses are obliquely oriented and nearly orthogonal. The orientation of the major axis for the CPs >0.50 ellipse is 53.71° with a bootstrapped 95% confidence interval of [34.92° 68.60°], indicating that it is elongated along quadrants I and III. The orientation of the major axis for the CPs <0.50 ellipse is 153.67° with a bootstrapped 95% confidence interval of [142.53° 165.04°], indicating it is elongated along quadrants II and IV. Thus, in CIP, neurons with CPs >0.50 tend to have slant and choice partial correlations of the same sign, whereas neurons with CPs <0.50 tend to have slant and choice partial correlations of opposite sign. The slant and choice partial correlations in CIP were not significantly correlated with each other overall (*r* = −0.17, *p* = 0.18), suggesting that slant and choice can have independent effects on neuronal responses (see Discussion).

In V3A, the mean (±SEM) CP for quadrants I and III (0.55 ± 0.03) was not significantly >0.50 (*N* = 9, Wilcoxon signed rank test, *p* = 0.09), but the mean CP for quadrants II and IV (0.44 ± 0.02) was significantly <0.50 (*N* = 14, Wilcoxon signed rank test, *p* = 0.02). This suggests that there was some tendency for the relative signs of the slant and choice partial correlations to predict CP in V3A. However, this trend was weak compared to CIP, as demonstrated by the 95% confidence ellipses for CPs > 0.50 and CPs < 0.50 in V3A. For both ellipses, the major axis is oriented approximately along the slant partial correlation axis (1.08° and −3.53° for CPs >0.50 and CPs <0.50, respectively), reflecting that the V3A responses were substantially more dependent on slant than choice.

### Time course of stimulus-related and choice-related activity in CIP and V3A

**Figure 8.**
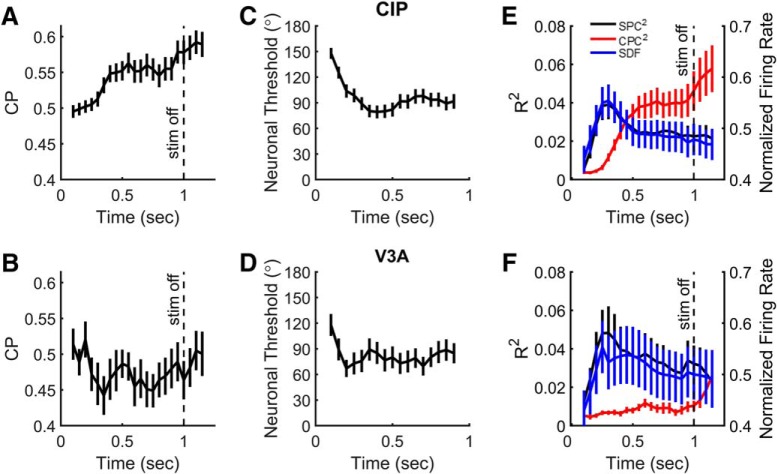
Time courses of choice probability, neuronal threshold, and partial correlations. ***A***, ***B***, Mean values of CP for CIP (***A***) and V3A (***B***) neurons as a function of time relative to stimulus onset. ***C***, ***D***, Mean neuronal thresholds (multiplied by $\sqrt{2}$) for CIP (***C***) and V3A (***D***) as a function of time. ***E***, ***F***, Mean SDFs (blue) as well as squared slant (SPC, black) and choice (CPC, red) partial correlations for CIP (***E***) and V3A (***F***) as a function of time. In all plots, analysis bins are 200 ms in duration, shifted every 50 ms starting at 100 ms. Each point is plotted in the center of the 200 ms time bin. Error bars denote SEM. Vertical dashed lines in ***A***, ***B***, ***E***, and ***F*** mark the end of the stimulus presentation. The last time bin is centered at 1150 ms, and thus extends approximately until the median choice time (1271 ms after stimulus onset).

Last, we examined the time course of CPs, neuronal thresholds, and partial correlations in CIP and V3A by computing these quantities within a series of 200 ms bins shifted every 50 ms. Average CP time courses are shown in Figure 8, *A* and *B*, for CIP and V3A, respectively. The mean CIP CP increased above baseline relatively late in the stimulus duration and remained elevated. The first time bin in which the mean (±SEM) CP (0.53 ± 0.01, *N* = 65 neurons) was significantly >0.50 was 350 ms (bin center) after stimulus onset (one-way ANOVA with multiple comparisons for *N* = 22 time bins, *p* < 0.05). The CP plateaued at ∼400 ms after stimulus onset and maintained this approximate level until the last time bin before stimulus offset (950 ms), at which point there was a further increase in CP, which may reflect additional choice-related activity and/or directionally selective saccade-related activity. The mean V3A CP was not significantly different from 0.50 in any time bin (one-way ANOVA with multiple comparisons for *N* = 22 time bins, *p* ≥ 0.05), but was slightly <0.50 throughout most of the stimulus duration. For comparison, mean CIP and V3A neuronal thresholds are shown in Figure 8, *C* and *D*, respectively.

The mean time courses for the spike density function (SDF; a measure of the average population response), squared SPC, and squared CPC are shown for CIP and V3A in Figure 8, *E* and *F*, respectively. The time courses of the squared slant partial correlations (black curves) are highly similar to the mean spike density functions (blue curves), with an early peak and smaller sustained values. In fact, the time course of the spike density function was highly correlated with that of the slant partial correlation in both areas (CIP, *r* = 0.93, *p* = 2.4 × 10^−10^, *N* = 22; V3A, *r* = 0.90, *p* = 1.9 × 10^−8^, *N* = 22). In CIP, the time course of the squared slant partial correlation peaked at ∼250–300 ms (bin centers), whereas the squared choice partial correlation increased later during the stimulus epoch (red curve). It was not until 450 ms (bin center) after stimulus onset that the squared choice partial correlation became significantly different from its initial value (one-way ANOVA with multiple comparisons, *p* < 0.05), further emphasizing that choice-related activity in CIP is substantially delayed relative to stimulus-related activity. Similar to the CP time course, the squared choice partial correlation plateaued until about the time of stimulus offset, at which point it increased further. In contrast, for V3A, the squared choice partial correlation remained close to zero throughout the stimulus duration, further reflecting that there was little to no choice-related activity in V3A. The squared choice partial correlation for V3A did, however, increase significantly above its initial value after stimulus offset, at the 1100 and 1150 ms time bins (one-way ANOVA with multiple comparison, *p* < 0.05). Given that the V3A CP was not significantly different from 0.50 in these same time bins (indicating that the increase in squared choice partial correlation was not linked to choices made in the direction of the preferred vs nonpreferred slant signs of the neurons, but instead up vs down choices), and, given that a previous study reported saccade-related activity in V3A (Nakamura and Colby, 2000), the increase in squared choice partial correlation following stimulus offset might be caused by directionally selective saccade-related activity.

## Discussion

We investigated correlations between 3D surface orientation perception and neuronal activity in areas V3A and CIP of the macaque monkey. Our results show that surface orientation is similarly discriminable based on V3A and CIP responses, and that neurons in the two areas are similarly sensitive to small slant variations. Together with anatomical data (Nakamura et al., 2001), these results suggest that V3A may, at least partially, underlie 3D orientation selectivity in CIP (Taira et al., 2000; Tsutsui et al., 2001; Rosenberg et al., 2013; Rosenberg and Angelaki, 2014b). Although stimulus-related activity was similar in the two areas, choice-related activity differed qualitatively. Specifically, choice-related activity during the slant discrimination task was prominent in CIP but largely lacking in V3A, implying a functional distinction between the areas. Together, these results suggest that both areas may contribute to 3D surface orientation processing, but that only CIP carries prominent 3D orientation choice-related signals.

### Comparison of stimulus-related and choice-related activity in CIP and V3A

The present results strongly agree with previous reports of 3D orientation selectivity in CIP (Taira et al., 2000; Rosenberg et al., 2013), and are consistent with previous studies implicating V3A in binocular disparity processing, 3D vision, and prehensile sensorimotor processing (Nakamura et al., 2001; Tsao et al., 2003; Anzai et al., 2011; Ban and Welchman, 2015; Goncalves et al., 2015). In both areas, 3D orientation preferences were shifted toward small slant preferences. This nonuniformity differs from our previous finding of a uniform distribution of 3D orientation preferences in CIP (Rosenberg et al., 2013), and may be a byproduct of extensive slant discrimination training about the frontoparallel plane. Based on 3D orientation tuning measured during passive fixation, the strength of selectivity was similar between the two areas (quantified using the SODI), though slightly greater in V3A than CIP. When slant tuning was measured during the slant discrimination task, V3A and CIP neurons were similarly sensitive to small slant changes, as evidenced by similar average neuronal thresholds. For some neurons in each area, neuronal thresholds were nearly as small as the behavioral threshold, suggesting that the monkeys may be less sensitive to changes in slant than is possible from an optimal decoding of the neuronal activity. Recent theoretical work suggests that suboptimal decoding and/or information-limiting noise correlations that introduce redundancy may cause behavioral thresholds to be only slightly smaller than individual neuronal thresholds (Moreno-Bote et al., 2014; Pitkow et al., 2015).

Although we found similar stimulus response properties in V3A and CIP, there was a stark difference in their choice-related activity. More than half of the CIP neurons had significant CPs, whereas only one V3A neuron had a significant CP. This difference suggests that CIP activity is functionally coupled with perceptual slant decisions, whereas V3A activity is not. However, given the small number of V3A neurons recorded in this study, this result should be considered preliminary. Additionally, the relationship between CIP activity and choice is not necessarily causal. Significant CPs could arise in a bottom-up manner (Britten et al., 1996; Haefner et al., 2013; Wimmer et al., 2015), but there is growing evidence that top-down (feedback) signals make important contributions to the presence of CPs (Nienborg and Cumming, 2009; Wimmer et al., 2015; Cumming and Nienborg, 2016; Kwon et al., 2016; Yang et al., 2016). Thus, observing significant choice-related activity does not necessarily imply a contribution to perceptual decisions, as reinforced by recent findings of dissociations between choice-related activity and reversible inactivation of brain areas. For example, neurons in the macaque ventral intraparietal area (VIP) have substantially greater CPs than those in the medial superior temporal area (MSTd) during a heading discrimination task (Gu et al., 2007, 2008; Chen et al., 2013), yet inactivation of MSTd impairs task performance, whereas inactivation of VIP does not (Gu et al., 2012; Chen et al., 2016). Similarly, neurons in the macaque lateral intraparietal area (LIP) show robust choice-related activity during motion discrimination, but inactivation of LIP does not impair task performance (Katz et al., 2016). A causal relationship between 3D surface orientation perception and CIP activity thus remains uncertain.

Previous work has shown that the 3D orientation tuning of CIP neurons is largely invariant to changes in the mean depth of the stimuli relative to the fixation plane, as well as the defining visual (i.e., perspective or stereoscopic) cue, suggesting that CIP neurons are sensitive to depth gradients (Taira et al., 2000; Tsutsui et al., 2001; Rosenberg and Angelaki, 2014b). In the present study, we did not have sufficient stimulus conditions to determine whether the slant selectivity of V3A neurons is also robust to changes in mean depth. Thus, we cannot rule out the possibility that the selectivity we observed in V3A reflects local disparity selectivity, given that local disparity within the receptive field changes as a function of slant in our stimulus. Indeed, an intriguing hypothesis is that our finding of robust CPs in CIP, but not in V3A, may be related to the extent to which these areas represent slant in a manner that is tolerant to variations in other cues (e.g., mean disparity). Specifically, it is possible that the lack of CPs in V3A results from a lack of tolerance to changes in mean disparity. We are currently conducting experiments to test this hypothesis directly.

### Dissociating the contributions of stimulus and choice to CIP and V3A activity

To dissociate the contributions of stimulus slant and choice to CIP and V3A responses, we computed partial correlations between these variables and the spike counts of individual neurons. In both areas, we found strong correlations between the stimulus and spike count. In contrast, correlations between choice and spike count were generally strong in CIP, but weak in V3A. This analysis validates the main CP finding; namely, that there was strong choice-related activity in CIP but not V3A. These results are reminiscent of a previous study, which found that V2, but not V1, neurons show significant choice-related activity during a disparity discrimination task (Nienborg and Cumming, 2006), despite the areas having similar disparity sensitivity. Thus, one potential explanation for these findings is that CPs observed in V2/CIP arise primarily from top-down signals that do not propagate back as strongly to V1/V3A. Another possibility, which is not mutually exclusive, is that the structure of correlated noise is different between V2/CIP and V1/V3A, reflecting that the appearance of CPs may depend on correlated noise (Shadlen et al., 1996; Nienborg and Cumming, 2006; Haefner et al., 2013) and perhaps particularly depend on correlated noise that is information limiting for the task at hand (Pitkow et al., 2015). An additional possibility, as noted above, is that CIP contains a more invariant representation of slant than V3A.

The pattern of slant and choice partial correlations observed in CIP may reflect a substantial top-down contribution to CPs. In a feedforward (bottom-up) scheme, it would be expected that stimulus and choice partial correlations would have the same sign, such that greater activity from a neuron constitutes evidence in favor of its preferred stimulus. In contrast, our CIP data show no significant relationship between slant and choice partial correlations (Fig. 7*A*). In other words, slant and choice signals are dissociated in CIP, similar to heading and choice signals in VIP (Zaidel et al., 2017). This dissociation may result from top-down choice-related signals that do not target CIP neurons according to their stimulus preferences.

It is also possible that some of the choice-related activity that we observed in CIP was due to directionally selective saccade-related activity. However, the time courses of CP and squared choice partial correlation suggest that saccade-related activity may be limited to the time period between stimulus offset and saccade execution. First, choice-related activity became significant ∼400 ms after stimulus onset (>800 ms before the median choice time). The choice-related activity then plateaued at an elevated value until about the time of stimulus offset. Second, there was a sharp increase in choice-related activity starting around stimulus offset, which may reflect a choice signal and/or directionally selective saccade-related activity. Together, these observations suggest that by restricting our analyses of choice activity to the stimulus presentation period, we largely isolated choice-related (rather than saccade-related) signals.

Last, we consider the relative timing of slant and choice signals. The time courses of slant-related signals in CIP and V3A were highly correlated with population-level spike density functions (Fig. 8). In CIP, the time courses of slant-related and choice-related signals differed substantially. Whereas the time course of the slant-related signals peaked ∼250 to 300 ms after stimulus onset, the choice-related signals did not become significant until ∼400 ms after stimulus onset. Late-onset choice-related activity has also been observed in other dorsal stream areas including the middle temporal area (Dodd et al., 2001) and anterior intraparietal area (Verhoef et al., 2010), and may be consistent with a top-down origin of choice signals in CIP, as suggested above based on the lack of correlation between slant and choice signals. Also consistent with this possibility, a previous study (Verhoef et al., 2012) found reaction times on the order of 250–350 ms in a convex–concave discrimination task, which is earlier than the start of significant choice-related activity that we found in CIP. Together, the present findings implicate V3A and CIP in 3D orientation processing, and suggest a qualitative distinction between the areas since only CIP showed choice-related activity during a fine slant discrimination task.

## Synthesis

Reviewing Editor: Siegrid Löwel, University of Goettingen Decisions are customarily a result of the Reviewing Editor and the peer reviewers coming together and discussing their recommendations until a consensus is reached. When revisions are invited, a fact-based synthesis statement explaining their decision and outlining what is needed to prepare a revision will be listed below. The following reviewer(s) agreed to reveal their identity: Peter Janssen, Igor Kagan.

Dear Authors,

both reviewers agree that you've submitted solid work on a very interesting topic, and that only minor revisions are required.

In general, your study advances the field by contributing to our understanding of how perceptual decisions about 3D stimuli are made in behaving macaques. Comparing choice-related activity in V3A and CIP, two areas in the dorsal stream, during perceptual discriminations, you describe 3D orientation tuning properties in area V3A. The main message is that both areas are equally selective for surface slant, and that only CIP but not V3A has significant choice-related activity. The study is well performed, the data are thoroughly analysed, the manuscript is well written and contains a lot of detailed information, the methods are sound and the conclusions are supported by the data. There are only few comments and suggestions for clarification to be considered for a revision.

A main conceptual issue concerns the possibility that choice selectivity in CIP might reflect saccade direction selectivity, rather than (or more likely in addition to) the perceptual choice. Given the proximity of the area CIP to oculomotor area LIP, it is not given apriori that CIP does not have cells with up/down saccade direction tuning. Since the monkeys reported the positive and negative slant with stereotypical up or down saccades, choice related and saccade preparation activity cannot be easily teased apart. In particular the presence of ‘incongruent’ cells with significant &lt;0.5 CP (and hence dissociated from slant preference) can in principle be interpreted as resulting from oculomotor preparation not related to specific perceptual choice. Therefore, the authors should present their arguments as to why they think it is not a confound.

Main other comments:

- The discrimination task was done with a fixed duration (1000 ms). Therefore, it is unknown when the decision about the direction of slant is made. The authors should refer to other studies such as Verhoef et al (2012) Neuron, where a reaction time version was used in a convex-concave discrimination task. Reaction times were on the order of 250 ms, so considerably shorter than the earliest rise in the CP in area CIP. This is consistent with a feedback signal after the decision has been made.

Minor comments:

Abstract: ‘many visual guided’ -&gt; visually guided

P1 line 14 ‘decision signals’: this may be confusing because the experiment did not measure a decision variable as in other studies using random dots. I would rephrase this in ‘choice-related signals’

P2 line 15 “3D curvature detection task” should be ‘3D curvature discrimination task’

P3 line 7: ‘recording grid’ should be ‘recording chamber’

P3 line 10: please add “to targets above and below the stimulus”

P4 line 1: when was the MRI taken? Did the authors visualize the grid positions in which they recorded?

Materials and Methods: p. 4, lines 2-6: I think an illustration (MRI or schematic drawing) would be helpful to better understand the recording trajectory, and the anatomical localization of recording sites in CIP and V3A (e.g. similar to Rosenberg et al. 2013).

P 10 line 11: was there any vergence response during stimulus presentation?

P 10 line 14: in this section and in subsequent sections, the authors should always indicate which time interval was used to calculate spikes and choice probabilities, because in one of the last figures they calculate the CPs as a function of time after stimulus onset.

P17 line 21: please specify which time bin this was: I assume the one from 250 to 450 ms after stimulus onset

P 21 line 2: here the authors should refer to other studies that found late CPs in dorsal stream areas: for example Dod, Kruger, Cumming and Parker (2001) J Neurosci, Verhoef et al. (2010) Curr Biol

Figure 1A: the illustration shows only 17 stimuli, but the actual number of stimuli is 25 slant-tilt combinations. May be add a note to the legend that only a subset of used stimuli are illustrated.

Figure 8: only 1 s stimulus presentation period is shown. It would be informative to see also the time course during the remaining reaction time period up to saccade initiation. Does the choice-related signal continue at the same level up to the saccade onset, or does it ramp up further?
